# Laser Annealing of P and Al Implanted 4H-SiC Epitaxial Layers

**DOI:** 10.3390/ma12203362

**Published:** 2019-10-15

**Authors:** Cristiano Calabretta, Marta Agati, Massimo Zimbone, Simona Boninelli, Andrea Castiello, Alessandro Pecora, Guglielmo Fortunato, Lucia Calcagno, Lorenzo Torrisi, Francesco La Via

**Affiliations:** 1 Dipartimento di Scienze Matematiche e Informatiche, Scienze Fisiche e Scienze della Terra (MIFT), Università degli studi di Messina, Viale F. Stagno d’Alcontres, 31-98166 Messina, Italy; lorenzo.torrisi@unime.it; 2 Consiglio Nazionale delle Ricerche-Istituto per la Microelettronica e i Microsistemi (CNR-IMM), VIII Strada, 5, 95121 Catania, Italy; marta.agati@imm.cnr.it (M.A.); massimo.zimbone@imm.cnr.it (M.Z.); simona.boninelli@imm.cnr.it (S.B.); guglielmo.fortunato@cnr.it (G.F.); francesco.lavia@imm.cnr.it (F.L.V.); 3 Consiglio Nazionale delle Ricerche-Istituto per la Microelettronica e i Microsistemi (CNR-IMM), Via del Fosso del Cavaliere, 100-00133 Roma, Italyalessandro.pecora@cnr.it (A.P.); 4 Dipartimento di Fisica e Astronomia (DFA), Università degli studi di Catania, Via S. Sofia 64, 95123 Catania, Italy; lucia.calcagno@ct.infn.it

**Keywords:** laser annealing, SiC, ion implantation, phosphorus, aluminum, Raman, photoluminescence, TEM, point defects, Metal Oxide Semiconductor Field Effect Transistor (MOSFET)

## Abstract

This work describes the development of a new method for ion implantation induced crystal damage recovery using multiple XeCl (308 nm) laser pulses with a duration of 30 ns. Experimental activity was carried on single phosphorus (P) as well as double phosphorus and aluminum (Al) implanted 4H-SiC epitaxial layers. Samples were then characterized through micro-Raman spectroscopy, Photoluminescence (PL) and Transmission Electron Microscopy (TEM) and results were compared with those coming from P implanted thermally annealed samples at 1650–1700–1750 °C for 1 h as well as P and Al implanted samples annealed at 1650 °C for 30 min. The activity outcome shows that laser annealing allows to achieve full crystal recovery in the energy density range between 0.50 and 0.60 J/cm^2^. Moreover, laser treated crystal shows an almost stress-free lattice with respect to thermally annealed samples that are characterized by high point and extended defects concentration. Laser annealing process, instead, allows to strongly reduce carbon vacancy (V_C_) concentration in the implanted area and to avoid intra-bandgap carrier recombination centres. Implanted area was almost preserved, except for some surface oxidation processes due to oxygen leakage inside the testing chamber. However, the results of this experimental activity gives way to laser annealing process viability for damage recovery and dopant activation inside the implanted area.

## 1. Introduction

4H-SiC popularity, due to its favourable properties for high-temperature and high-power applications, has recently grown and its expansion in semiconductor devices, and particularly in MOSFET technology, has begun. The realization of MOSFET selected doped areas, such as the source and body regions, are achieved through ion implantation. However, low diffusivity of mostly used dopants in SiC requires the use of multi-ion implantation with different energies and doses to achieve selected uniformly doped area. The realization of MOSFET’s n-type source region occurs through phosphorus ion implantation, while the p-type body region is obtained using Al. 

The use of these processes involves the generation of a considerable implantation defectiveness, which is only partially recovered by conventional thermal treatments. Moreover dopant activation is strongly limited both by dopant solid solubility, and by the attainable annealing temperature in conventional high temperature (HT) steps. Thermal annealing is also involved in V_C_ generation under standard thermal equilibrium processes so that new non equilibrium methods based on short time annealing duration are required. A comprehensive description of thermal annealing dynamics is provided by transient model activation [1], which states that the initial activation speed for both donor and acceptor impurities is extremely high and decrease rapidly with time. Indeed, rapid thermal annealing systems, are able to obtain high dopant incorporation eluding the usual impurity deactivation due to solid solubility lowering during cooling ramps. In particular, laser annealing technique counts on ramps as high as 10^9^ K/s and allows to obtain much higher temperatures than conventional processes.

After first Hishida [2] and Ahmed’s [3] laser annealing attempts on 4H-SiC, Tanaka [4] pointed out that 4H-SiC melt phase annealing was not a suitable post implant treatment due to the impossibility of obtaining full 4H-SiC epitaxial regrowth. Consequently excimer laser pulses under sub-melting condition were proposed to perform a sequence of rapid thermal annealings in the ns time regime. Boutopoulos [5] confirmed the viability of this method carrying out a structural micro-Raman analysis of multishot laser annealing on Al implanted 4H-SiC. He observed how Nd:Yag (355 nm) multiple irradiation method was able to significantly recover reflectivity signal intensity in the Restrahlen band. However, TEM analysis showed the formation of polycristalline structure after laser annealing process. Similar results are stated by Mazzamuto [6], who claims to obtain full epitaxial regrowth from SiC melting phase, though TEM analysis show polycrystalline lattice recovery after ion implantation.

The aim of this work is to offer a pioneering characterization of laser annealing effects on implanted SiC with particular concern to phase separation energy thresholds related to SiC non-congruent melting. We perform an effective study of the exploitable XeCl laser energies, as well as an atomic scale characterization that confirms the complete 4H polytype maintenance under sub-melting regime.

## 2. Materials and Methods 

Experimental activity was conducted on SiC wafer supplied by Cree with a nitrogen concentration of 10^18^ N/cm^3^. A 6 μm epitaxial layer was then grown along the (0001) 4° off-axis direction through low pressure hot wall chemical vapor deposition. Source P ion implantation was performed at 500 °C with energies between 30 and 200 keV and fluences ranging from 10^13^ to 10^14^ cm^−2^ in order to obtain an almost uniform doped layer, 200 nm thick, with a concentration of 10^20^ cm^−3^. In the double implanted sample a further multistep implantation was performed with Al doses between 10^12^ and 10^13^ cm^−2^ at 400 °C. The wafer was then cut into several squares using a diamond tip to obtain different samples on which we performed the tests. In order to eliminate any impurities on the surface, the samples were subsequently bathed in isopropyl alcohol and dried by compressed air jet. Laser treatments were carried out through a LPX 300 XeCl pulsed excimer laser (λ = 308 nm, Lambda Physics, Göttingen, Germany) with 40 Hz repetition rate, whose beam intensity is tuned by a MicroLas Lasersystem attenuator unit, which accomplishes the variation of laser pulse energy by almost two order of magnitude in steps of 1%. The beam is then sent to an homogenizer, which divides the laser beam into many sections (splitter optical element), superimposing them in a plane, called the focal plane, so that the final beam assumes the desired dimensions (condenser optical elements). The samples were so irradiated by a 1 mm × 20 mm uniform intensity profile radiation inside a chamber under helium atmosphere at a pressure of 1 × 10^−2^ mbar. Substrate holder inside the chamber was heated at a temperature of 580 °C and a mobile stage allowed to implement an irradiation process until 1000 shots/point. On the other hand, isochronical (1 h) thermal annealings were carried out at 1650 °C, 1700 °C, 1750 °C for P implanted samples, while a sample subjected to double implantation was annealed at 1650 °C for 30 min. The effectiveness of laser treatments was verified by micro-Raman and photoluminescence analysis, carried out with a He-Cd laser source (λ = 325 nm) and a LabRAM HR spectrofluorimeter (Horiba Jovin Yvon, Kyoto, Japan) with a 1800 l/mm grating. Structural investigation was performed using TEM microscopy on a 2010F Jeol microscope (Tokyo, Japan) equipped with a Gatan image filter for electron energy loss spectroscopy (EELS). 

## 3. Results and Discussion

Laser thermal processing was first inspected by Raman spectroscopy. This technique is widely used for SiC characterization since it is non-destructive, requires no special sample preparation and presents high sensitivity on structural changes, so that a survey methodology to verify crystal damage recovery can be obtained. Indeed, it is known that ion implantation process generates damage inside the crystal, and although implantation is carried out at 550 °C to avoid lattice amorphization, a decrease of first order Raman peaks intensities has been observed proportional to implantation dose [7]. 

With the increase in the number of laser shots it is possible to observe the recovery of the 4H-SiC characteristic phonon modes: E_2_(TA) at 205.5 cm^−1^, A_1_(LA) at 612.1 cm^−1^, E_2_(TO) at 776.0 cm^−1^, E_1_(TO) at 798.6 cm^−1^ and A_1_(LO) at 967.3 cm^−1^. The increase in the intensity of these Raman peaks as a function of the radiation energy density shows how the laser is able to produce an annealing effect to recover sample crystallinity.

**Figure 1:**
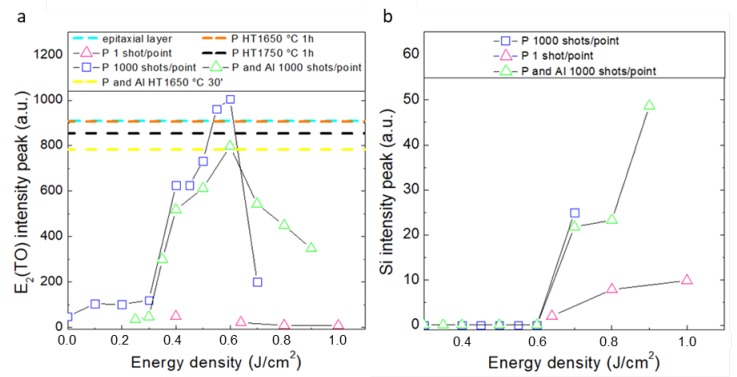
(**a**) Raman E_2_(TO) mode and (**b**) Si Raman peak intensity peak vs laser energy density for single source implant and double source and body implants.

Transverse optic E_2_(TO) mode signal intensities were extrapolated from Raman spectra and are reported in Figure 1a. In the case of 1 shot/point, crystal recovery is weak compared to as implanted sample signal. However, when the number of shots is increased up to 1000 shots/point, it can be seen that both samples with single and double implant exhibit E_2_(TO) signal intensity enhancement and existence of 3 regions is found. Up to 0.30 J/cm^2^ E_2_(TO) peak intensity remains low. Between 0.30 J/cm^2^ and 0.60 J/cm^2^ the TO mode signal sharply rises up to its maximum, until the onset of phase separation, where 4H-SiC Raman signal E_2_(TO) goes down and crystalline silicon peak at 520 cm^−1^ together with low carbon G band at 1592 cm^−1^ are detected. In thermally annealed samples, the attempt to increase the fraction of dopant activated by increasing the annealing temperature from 1650 °C to 1750 °C produces a 15% reduction in E_2_(TO) signal intensity and broadening from 5.3 to 8.1 cm^−1^, due to the increase in thermal generated defectiveness not only within the projected range of the system but throughout the entire sample, so that worsening of crystallinity is therefore deducible.

Raman signals coming from samples subjected to double implantation are slightly lower than the single implantation one. In this case, in fact, the presence of a second 700 nm deep layer involves an increase of damage and consequently a lowering of the Raman peak intensities.

When the sample is irradiated with an energy above the 0.60 J/cm^2^ threshold, Raman spectrum shows a further peak at 520 cm^−1^. This new vibrational frequency is associated with crystalline Si layers deposited on surface due to phase separation occurring when SiC melting temperature is reached. Figure 1b shows that the Si peak intensity increases linearly with the energy density exceeding 0.60 J/cm^2^ for both types of samples analyzed.

The identification of this energy threshold is consistent with the results coming from numerical solution of heat flow equation and expressed as

(1) $$\frac{\partial T}{\partial t}=\frac{\alpha }{\rho C_{p}}I\left(z,t\right)+\frac{1}{\rho C_{p}}\frac{\partial }{\partial z}\left(k\frac{\partial T}{\partial z}\right)$$

(2) $$I\left(z,t\right)=I_{0}\left(t\right)\left(1-R\right)\exp\left(-\alpha z\right)$$

where *T* represents the absolute temperature, *ρ* is sample density, *C_p_* the specific heat, *k* is thermal conductivity, *I*_0_ is laser intensity at the surface, *R* is surface reflectivity, *α* the optical absorption, *z* the depth from surface, *I* (*z*,*t*) laser intensity. According to Equations (1) and (2), the melting threshold of as-implanted SiC irradiated with 30 ns XeCl pulse length stands over 0.60 J/cm^2^ [8], while to induce phase separation in crystalline SiC a laser energy equal to 1 J/cm^2^ is required. This prediction is based on the assumption that, when the implanted layer thickness exceeds 200 nm, optical parameters are in reasonable agreement with that of bulk amorphous SiC.

**Figure 2:**
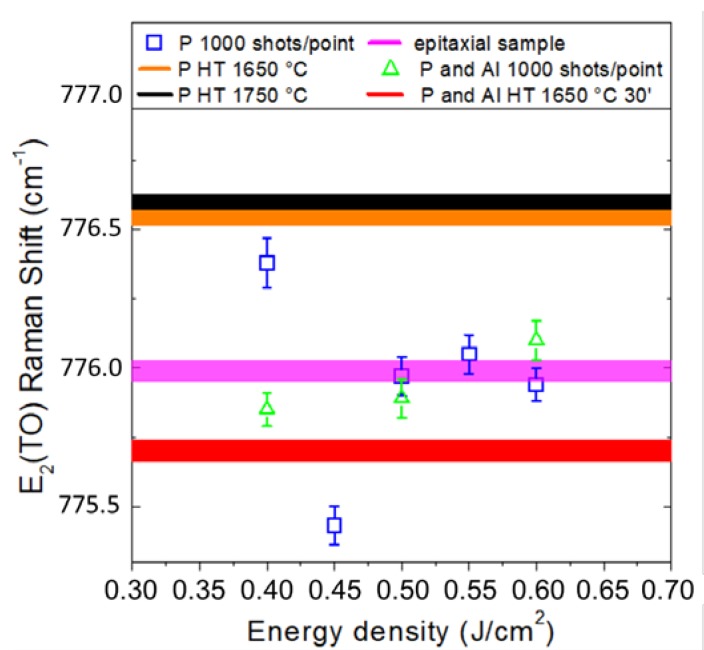
Raman E_2_(TO) vibrational mode frequency vs Energy density for single source implant and double source and body implants.

Samples subjected to thermal annealing also show a deviation from the nominal 4H-SiC vibrational frequency between 1650 °C and 1750 °C (Figure 2). The effect arising from such a high thermal budget results in the generation of a compressive (P implanted) and tensile (P and Al implanted) stress particularly assessable along the planar component, since the E_2_(TO) modes have an atomic displacement perpendicular to the c axis [9]. From the equations

(3) $$\Delta \omega_{E_{2}}=\left(2a_{E2}+b_{E2}\right)P=3.59P$$

(4) $$a_{E2}=-1.55\;{\mathrm{cm}}^{-1}/\mathrm{GPa}$$

(5) $$b_{E2}=-0.74\;{\mathrm{cm}}^{-1}/\mathrm{GPa}$$

where $\Delta \omega_{E_{2}}$ is the phonon frequency shift, $a_{E2}$ and $b_{E2}$ are the phonon deformation potentials and P is the in plane pression, it is therefore possible to provide an estimate of the stress, which in the case of thermally annealed samples ranges from 130 to 172 MPa increasing with temperature. The usual thermal processes generate a strong stress contribution that is due to multiple factors. In small part this is due to the incorporation of the P or Al dopant responsible for opposing stress contributions and to the shear stress between the implanted area and the underlying epitaxial layer, while the predominant role is related to the creation of a high concentration of both point and extended defects in the implanted region [10]. 

Samples subjected to laser annealing show much smaller stress values. Especially in high recovery ranges between 0.50 and 0.60 J/cm^2^, vibrational frequencies close to epitaxial mode were detected and calculated stress does not exceed 50 MPa, so that almost stress-free samples resulted from laser annealing process.

**Figure 3:**
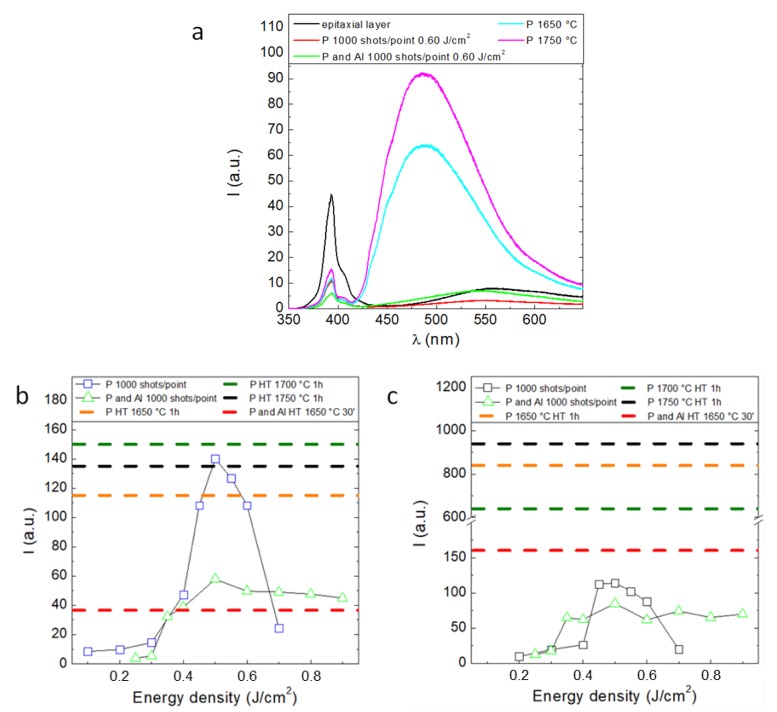
(**a**) PL spectra or single source implant and double source and body implants (**b**) band to band and (**c**) intra-band-gap PL intensity peak vs. laser energy density.

The presence of an important crystal recovery effect is also visible by mean of the photoluminescence analysis. Figure 3a reveals how progressive irradiation at 1000 shots/point produces 4H-SiC band-gap signal recovery at 390 nm. The intensity value of the band–band peak is equal to that of the sample subjected to annealing for 1 h at 1650 °C (see Figure 3b). Thermally annealed samples exhibit a large peak centred at 490 nm with 175 nm full width at half maximum (FWHM), which can be related to the generation of a high defectiveness concentration and in particular to Vc generated in the implanted region and in the epitaxial layers [11,12]. In addition, interstitial aggregates present within the implanted layers provide an emission contribution to wavelengths corresponding to intra-band-gap emission signal. The PL results coming from laser annealed samples confirm the crystalline recovery trend as appeared from the Raman analysis. At 490 nm no large region of defectiveness is observed within the samples subjected to laser annealing. As shown in Figure 3b, these samples have, in fact, a band peak at 390 nm growing noticeably starting from 0.40 J/cm^2^, reaching its maximum at 0.50 J/cm^2^ and falling slightly at 0.60 J/cm^2^, beyond which phase separation is detected. At 0.50 J/cm^2^ the 390 nm peak exceeds the emission intensity of the sample subjected to annealing at 1750 °C. At 0.60 J/cm^2^ the 390 nm peak intensity tends to decrease due to progressive enhancement of laser surface effects. The same trend is reported for intra-band-gap peak emission (Figure 3c). However, laser annealing leads to a 60 nm shift of intra-band-gap radiative recombination signal by 60 nm with respect to the corresponding thermal annealing values, making the emission peak coincide with the epitaxial layer PL emission at 550 nm. Moreover, intra-band-gap emission peak intensity is definitely lower than that detected for thermally annealed samples: The ratio between band to band and intra-band-gap peaks in P implanted samples states on 1.2 between 0.50 and 0.60 J/cm^2^ and decreases to 0.45 once the sample undergoes phase separation at 0.7 J/cm^2^. Thermally annealed samples show much lower values attested at 0.1. Therefore, the deviation between the values obtained for laser and thermal annealing highlights how out of thermal equilibrium crystal recovery leads to a concentration of defects lower by a factor of 8–9. The PL analysis on double implanted samples confirms this trend. However, in this case the addition of Al further implantation to achieve the 800 nm thick body region results in sample signal attenuation due to the increase in defectiveness along the He-Cd laser penetration path. However it is worth noting that from 0.50 J/cm^2^ the laser annealing effect on P and Al implanted samples leads to a higher band to band peak response compared to the corresponding thermally annealed samples.

**Figure 4:**
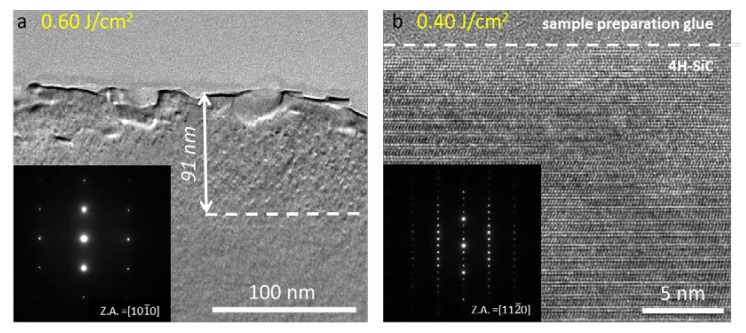
(**a**) XTEM Bright field image of the 0.60 J/cm^2^ laser annealed source implant. The inset shows the 4H-SiC diffraction pattern under [11$\bar{1}$0] zone axis. (**b**) HRTEM of the source implant sample subjected to 0.40 J/cm^2^ laser fluence showing periodic ABAC stacking sequence acquired in correspondence of the surface under the [11$\bar{2}$0] zone axis. The inset reports the related 4H-SiC diffraction pattern.

**Figure 5:**
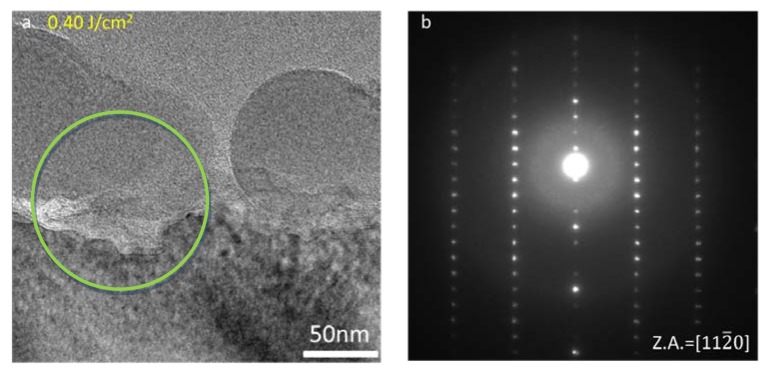
(**a**) Bright field X TEM image of 0.40 J/cm^2^ source implant annealed sample surface. Green circle indicates the area where (**b**) selected area diffraction (SAD) has been acquired.

Bright field TEM analyses were performed to structurally characterize the effects due to laser irradiation. Several extended defects are detected inside the implanted area, as shown in Figure 4a in the case of the 0.60 J/cm^2^ laser annealed source implant sample. The presence of these defects allows to quantify the portion of implant survived to laser interaction. It was possible to estimate that within the 0.40 J/cm^2^ irradiated sample an implanted area of almost 130 nm was preserved (image not shown), while with the increase of laser energy density this fraction is thinned to 90 nm (see Figure 4a). However, the cross section confirms the data obtained with Raman spectroscopy. In fact, in the inset of Figure 4a, the diffraction pattern acquired on the [10$\bar{1}$0] zone axis shows the full crystallinity of the implanted area. From Figure 4a we can see that laser interaction gives rise to a certain degree of surface misalignment, although the crystal is kept under the 4H polytype. Figure 4b shows the 0.40 J/cm^2^ irradiated sample HRTEM. It can be highlighted that inspected region maintained the ABAC stacking characteristic of the 4H polytype under the [11$\bar{2}$0] zone axis up to the top. It is evident that the crystal did not undergo any melting process that would have produced 3C-SiC layer on surface [13] combined with the SiC columnar regrowth from the melt/solid interface [6]. However, since SiC (0001) is characterized by a relatively important surface energy [14], high temperature treatments commonly promote surface desorption of silicon atoms from the surface and consequent micro-step formation. Nevertheless, the presence of a further contribution to surface misalignment on many samples is visible in Figure 5a. Spherical aggregates with average size of 131 nm and with 21% of surface coverage are, in fact, detected on the sample. A first structural analysis was carried out by performing a selected area diffraction (SAD) relative to the area marked by the green circle in Figure 5a. Figure 5b highlights the presence of the resulting 4H-SiC diffraction pattern under the zone axis [11$\bar{2}$0], as well as the presence of circles indicating amorphous material within the investigated area.

**Figure 6:**
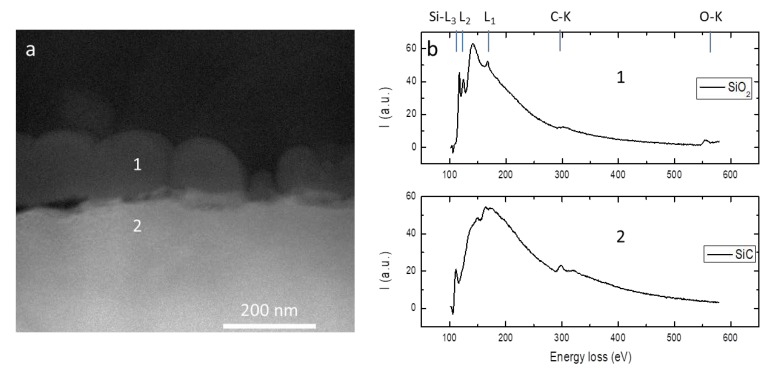
(**a**) STEM image of 0.40 J/cm^2^ treated source implant surface. (**b**) Electron energy loss spectroscopy (EELS) spectra coming from surface aggregates and from implanted area.

To perform an elemental analysis the sample was analyzed using STEM-EELS spectrum imaging. In Figure 6b we reported the EELS spectra acquired in correspondence of the region 1 and 2 of the sample, as indicated in Figure 6a. In the EELS spectrum corresponding to region 1, we can easily recognize the characteristic SiO_2_ spectrum, where it is possible to identify the Si-L_1_, Si-L_2_,_3_ and O-K edges. The less intense signal coming from C is due to the epoxy glue employed during sample preparation, composed of (C_6_H_6_O·CH_2_O)_x_ and C_3_H_4_N_2_. The EELS spectrum corresponding to region 2, instead, shows the typical SiC signal coming from bulk SiC. 

Therefore, once identified surface aggregates as SiO_2_ compounds, it is possible to attribute their formation to the presence of residual O in the chamber as an etching source. Studies of the reaction between O and SiC [15] allow, in fact, to state that the interaction between O and SiC at high temperatures is not only present in the form of passive oxidation, but at low pressures it results in phenomena of active oxidation, leading to surface etching and contextual important role in C surface segregation. It can therefore be inferred that O presence in chamber may have played a role in superficial roughness while etching contributions of the sample are due to the local temperature rising at the surface layers and to the presence of photochemical effects inherent to the interaction with the environment inside the chamber during irradiation.

Preliminary results from the Circular Transfer Length Method (CTLM) characterization allowed to establish resistivity values between 1.69·10^−2^ and 2.17·10^−2^ Ω·cm. Associating the doping profile coming from Secondary Ion Mass Spectroscopy (SIMS) analysis (not shown here) with XTEM results, it is possible to extract the average value of total P concentration equal to 2.64·10^19^ P/cm^3^ in the source region. By extrapolating the concentration from the resistivity plot as a function of doping for an epitaxial layer [16], it can be assumed that the active dopant concentration reaches a value between 2.5 and 5.6·10^18^ e/cm^3^. However, these activation values are actually underestimated because they take into account higher electron mobility than those present in the implant end-range and prelude to a greater activation. Therefore, once tested the effectiveness in crystalline recovery, the current challenge in the field of laser annealing goes through strategies aimed at improving surface protection and limiting roughening factors.

## 4. Conclusions

In this article the results of laser annealing related to implanted epitaxial layers with single P implantation and double P and Al implantations were compared with the single thermal annealing treatment. From the work presented here it is clear that the XeCl multi-laser pulses system is able to recover implantation damage. By observing the intensity variation of Raman phonon modes it was possible to plot crystal recovery trend as a function of energy density, from sub melting regime to phase separation. Compared to the samples subjected to thermal annealing, laser treated samples present a lower defect optical signal by a factor up to 8–9 compared to thermally annealed samples. This shows that the laser pulse acting in a non-equilibrium regime and taking advantage of rapid annealing and cooling ramps is able to recover implantation damage generating a lower point defects concentration. Surface etching phenomena are due both to Si atoms desorption already known in conventional thermal annealing and to oxidation phenomena occurring inside the chamber in the presence of O. It is possible to affirm that laser annealing constitutes, with suitable precautions, a viable way as a post-implant thermal process.
